# Supplementary material for: Self-identity explains better breastfeeding intention of ethnic pregnant mothers of Western Nepal: Extending the theory of planned behavior
Source: PLoS One. 2024 Oct 11;19(10):e0312010. doi: 10.1371/journal.pone.0312010 (PMC11469527; doi:10.1371/journal.pone.0312010)
Supplement: S1 File — Constructs and measurement. (DOCX) [file pone.0312010.s001.docx]

**Title: Self-identity explains better breastfeeding intention of ethnic pregnant mothers …**

S1.Table 1-16. Constructs and measurements

S1.Table 1. Breast feeding knowledge inventory (English)

| **SN** | **Question** | **Response** | **Score** |
| --- | --- | --- | --- |
| B1 | Have you heard about colostrum milk? | Yes  No | 1  0 |
| B2 | If yes, what is colostrum milk? | ▪Correct description (only breastfeed with medication upto six months)  ▪Incorrect description (also others…in addition to breastfeed, medication) | 1  0 |
| B3 | Do you Know when to initiate to breastfeeding by the mother? | ▪Within 1 hour after birth  ▪After 1 hour of delivery  ▪Don’t know | 1  2  3 |
| B4 | Have you heard of exclusive breast feeding? | ▪Yes  ▪NO | 1  0 |
| B5 | Assess the definition of exclusive breastfeeding given by mother. | ▪Correct definition  ▪Incorrect definition | 1  0 |
| B6 | What are the advantages of breastfeeding? | ▪Yes  ▪NO | !  ) |
| B7 | If yes, to whom is it advantageous? | ▪to mother only  ▪to infant only  ▪both to mother and infant | !  @  # |

S1.Table 2. Breast feeding knowledge inventory (Nepali)

| **qm= ;=** | **k\|Zg** | **;f+s]lts k\|sf/** | **cFs** |
| --- | --- | --- | --- |
| B1 | तपाँइले बच्चालाई खुवाउने विगाैति दुधको बारेमा सुन्नुभएको छ ? | **छ (यदी छैन भने B3 मा जानुहोस् )**  **छैन** | !  ) |
| B2 | यदि छ भने विगाैति दुध भनेको के हो? | **सहि उत्तर**  **गलत उत्तर** | !  ) |
| B3 | बच्चालाई दुध चुसाउन शुरुवात कुन बेलामा गर्नुपर्छ s] तपाइलाई थाहा छ ? | **बच्चा जन्मिएको १ घण्टा भित्र**  **बच्चा जन्मिएको १ घण्टा पछाडि**  **थाहा छैन** | !  @  # |
| B4 | तपाँइले ljz]if :tgkfgको बारेमा सुन्नुभएको छ? | **छ**  **छैन (यदी छैन भने** B6 **मा जानुहोस् )** | !  ) |
| B5 | यदि छ भने ljz]if स्तनपान u/fpg' भनेको के हो ? | **सहि उत्तर**  **गलत उत्तर** | !  ) |
| B6 | तपाइलाई स्तनपानका फाइदाहरु थाहा छ ? | **छ**  **छैन (यदी छैन भने C1 मा जानुहोस् )** | !  ) |
| B7 | यदि छ भने यसले कसलाई फाइदा गर्छ ? | **आमालाई मात्र फाइदा गर्छ**  **बच्चालाई मात्र फाइदा गर्छ**  **आमा र बच्चा दुवैलाई फाइदा गर्छ** | !  @  # |

S1. Table 3. Breast feeding intention score (English)

| **Q.N** | **Questions** | **Category of coding** | **Scoring** |
| --- | --- | --- | --- |
| C1 | Do you think you are well prepared for breastfeeding? | Completely unsure  Unsure  Neither sure nor unsure  Sure  Completely sure | 1  2  3  4  5 |
| C2 | Do you intend to give any prelacteal fluid to your baby after birth**?** | Straight ‘no’  No  Don’t know  Yes  Straight ‘yes’ | 1  2  3  4  5 |
| C3 | What is the probability of you breast feed your child? | Highly impossible  Impossible  Neither possible nor impossible  Possible  Highly possible | 1  2  3  4  5 |
| C4 | How much are you committed to breast feed your baby? | Highly uncommitted  Uncommitted  Neither committed nor uncommitted  Committed  Highly committed | 1  2  3  4  5 |
| C5 | How much are you determined to breast feed your baby? | Highly undetermined  undetermined  Neither determined nor undetermined  Determined  Highly determined | 1  2  3  4  5 |

S1. Table 4. Breast feeding intention score (Nepali)

| **qm= ;=** | **k\|Zg** | **;f+s]lts k\|sf/** | **cFs** |
| --- | --- | --- | --- |
| C1 | के तपाँइ आफ्नो बच्चालाई स्तनपान गराउन tof/ x'g'हुन्छ? | **निस्चित गर्दिन्**  **सायद गर्दिन**  तटस्थ  **सायद गर्छु**  **निस्चित गर्छु** | !  @  #  $  % |
| C2 | तपाँइ आफ्नो बच्चालाई कत्तिको स्तनपान गराउन चाहानु हुन्छ | kSsf **चाहान्न**  **चाहान्न**  **तटस्थ**  **चाहान्छु**  kSsf **चाहान्छु** | !  @  #  $  % |
| C3 | तपाँइn] आफ्नो बच्चालाई स्तनपान गर्ने सम्भावना कस्तो छ ? | **धेरै असम्भावना छ**  **असम्भावना छ**  **तटस्थ**  **सम्भावना छ**  **धेरै सम्भावना छ** | !  @  #  $  % |
| C4 | तपाँइ आफ्नो बच्चालाई स्तनपान गराउन कत्तिको प्रतिबद्द हुनुहुन्छ < | **अति अप्रतिबद्द छु**  **अप्रतिबद्द छु**  **तटस्थ**  **प्रतिबद्द छु**  **अति प्रतिबद्द छु** | !  @  #  $  % |
| C5 | तपाँइ आफ्नो बच्चालाई स्तनपान गराउनु k5{ eGg] s'/fdf कत्तिको b[9 x'g'x'G5< | **अति** clglZrt  clglZrt  **तटस्थ**  lglZrt  **अति** lglZrt | !  @  #  $  % |

S1. Table 5. Breast feeding attitude score (English)

| **Q.N** | **Questions** | **1 (Strongly disagree)** | **2 (Disagree)** | **3 (Neutral)** | **4 (Agree)** | **5 (Strongly agree)** |
| --- | --- | --- | --- | --- | --- | --- |
| D1* | If I feel discomfort when breast feeding, I will shift to bottle feeding. | 1 | 2 | 3 | 4 | 5 |
| D2 | I will breastfeed within 1 hour of delivery even if there is any discomfort. | 1 | 2 | 3 | 4 | 5 |
| D3* | I won’t give any supplemental food until baby reaches two years. | 1 | 2 | 3 | 4 | 5 |
| D4* | I won’t breast feed if anybody dishonors me. | 1 | 2 | 3 | 4 | 5 |
| **SN** | **Statements** | **Options** | | | | **Score** |
| D5 | I feel….when I breastfeed my baby. | Very sad  Sad  Neutral  Happy  Very happy | | | | 1  2  3  4  5 |
| D6 | I feel…..when I breastfeed my baby. | Very sick  Sick  Neutral  Healthy  Very healthy | | | | 1  2  3  4  5 |
| D7 | I feel…..when I breastfeed my baby. | Very hatred  Hatred  Neutral  Attractive  Very attractive | | | | 1  2  3  4  5 |
| D8 | I feel…..when I breastfeed my baby. | Very discomfortable  Discomfortable  Neutral  Comfortable  Very comfortable | | | | 1  2  3  4  5 |
| **SN** | **Statements and scores** | **Strongly disagree** | **Disagree** | **Neutral** | **Agree** | **Strongly agree** |
| D9* | As part of tradition, prelacteal fluid must be fed to the newborn before breastfeeding. | 1 | 2 | 3 | 4 | 5 |
| D10* | Exclusive breastfeeding to babies for 6 months is not a good practice. | 1 | 2 | 3 | 4 | 5 |
| D11 | Colostrum Milk protects the baby from infections. | 1 | 2 | 3 | 4 | 5 |
| D12 | Breast milk is cheaper than infant formula. | 1 | 2 | 3 | 4 | 5 |

S1. Table 6. Breast feeding attitude score (Nepali)

| **qm=;=** | | **k\|Zg -cFs_** | | **अति असहमत** | | **असहमत** | | **तटस्थ** | | **सहमत** | **अति सहमत** |
| --- | --- | --- | --- | --- | --- | --- | --- | --- | --- | --- | --- |
| D1* | | यदी मलाई स्तनपान गर्दा असुबिधा हुन्छ भने म बोटल दुधले प्रतिस्थापना गर्नेछु **.** | | ! | | @ | | # | | $ | % |
| D2 | | म मेरो नवजात सिसुलाई प्रसब पछी असुबिधा महसुस भए पनि जन्मेको एक घण्टा भित्रै स्तनपान गराउँछु **.** | | ! | | @ | | # | | $ | % |
| D3* | | म मेरो बच्चालाई दुई बर्ष सम्म थप खानेकुरा दिनेछैन **.** | | ! | | @ | | # | | $ | % |
| D4* | | स्तनपान गरको बेलामा कसैले मलाई अपमन गर्छ भने म मेरो बच्चालाई स्तनपान गराउँदिन **.** | | ! | | @ | | # | | $ | % |
| **qm=;=** | **k\|Zg** | | **;f+s]lts k\|sf/** | | | | | | | | **cFs** |
| D5 | मेरो बच्चालाई स्तनपान गराउँदा मलाई … dxz'; x'G5 . | | अति b'Mख  b'Mख  तटस्थ  सुख  अति सुख | | | | | | | | !  @  #  $  % |
| D6 | मेरो बच्चालाई स्तनपान गराउँदा मलाई … dxz'; x'G5 . | | अति अस्वस्थ  अस्वस्थ  तटस्थ  स्वस्थ  अति स्वस्थ | | | | | | | | !  @  #  $  % |
| D7 | मेरो बच्चालाई स्तनपान गराउँदा मलाई … dxz'; x'G5 . | | अति घृणित  घृणित  तटस्थ  आकर्षक  अति आकर्षक | | | | | | | | !  @  #  $  % |
| D8 | मेरो बच्चालाई स्तनपान गराउँदा मलाई…dxz'; x'G5 . | | अति असुबिधा  असुबिधा  तटस्थ  सुबिधा  अति सुबिधा | | | | | | | | !  @  #  $  % |
| **qm=;=** | **k\|Zg tyf cFs** | | **अति असहमत** | | **असहमत** | | **तटस्थ** | | **सहमत** | | **अति सहमत** |
| D9* | प्राचिन रितिरिवस अनुसार नवजात शिशुलाई जन्मिने बित्तिकै स्तनपान भन्दा अगाडि अरु केहि झोल अथवा तरल पर्दाथ खुवाउनु पर्छ कत्तिको सहमत हुनुहुन्छ? | | ! | | @ | | # | | $ | | % |
| D10* | ६ महिना हुँदा सम्म बच्चालाई बिशेष स्तनपान गराउनु राम्रो होइन । कत्तिको सहमत हुनुहुन्छ ? | | ! | | @ | | # | | $ | | % |
| D11 | बिगाैति दुधले बच्चालाई सङक्रमण बाट बचाउँछ । कत्तिको सहमत हुनुहुन्छ? | | ! | | @ | | # | | $ | | % |
| D12 | आमाको स्तनपान बजारमा पाउने दुध वा पाउडर दुध भन्दा सस्तो पर्छ । कत्तिको सहमत हुनुहुन्छ? | | ! | | @ | | # | | $ | | % |

S1. Table 7. Breast feeding subjective norm score (English)

| SN | Statements | Options | Score |
| --- | --- | --- | --- |
| E1* | People who are important to me think I should… | Surely not breast feed  Not breast feed  Neutral  Breast feed  Surely breastfeed | 1  2  3  4  5 |
| E2 | People who are important to me allow me to breastfeed by baby. | Strongly disagree  Disagree  Neutral  Agree  Strongly agree | 1  2  3  4  5 |

S1. Table 8. Breast feeding subjective norm score (Nepali)

| **qm= ;=** | **k\|Zg** | **;f+s]lts k\|sf/** | **;+s]t** |
| --- | --- | --- | --- |
| E1* | जो मेरा लागि महत्तोपूर्ण ब्यक्तिहरु छन् उनिहरुले सोच्छन् कि मैले … | निश्चित रूपमा स्तनपान गर्नु हुँदैन  स्तनपान गर्नुहुँदैन  तटस्थ  स्तनपान गर्नुपर्छ  निश्चित रूपमा स्तनपान गर्नुपर्छ | !  @  #  $  % |
| E2 | जो मेरा लागि महत्तोपूर्ण ब्यक्तिहरु छन् उनिहरुले मलाई आफ्नो बच्चालाई स्तनपान गराउन सहमतL दिन्छन् . | अति असहमत  असहमत  तटस्थ  सहमत  अति सहमत | !  @  #  $  % |

S1. Table 9. Perceived breastfeeding control score (English)

| SN | Statements | Options | Score |
| --- | --- | --- | --- |
| F1 | When I breastfeed my baby, it is …..for me. | Very difficult  Difficult  Neutral  Easy  Very easy | 1  2  3  4  5 |
| F2 | When I breastfeed my baby, something will happen which will stop me from breastfeeding. | Strongly disagree  Disagree  Neutral  Agree  Strongly agree | 1  2  3  4  5 |
| F3 | I am confident that I can breastfeed when it is needed to my baby. | Strongly disagree  Disagree  Neutral  Agree  Strongly agree | 1  2  3  4  5 |

S1. Table 10. Perceived breastfeeding control score (Nepali)

| **qm= ;=** | **k\|Zg** | **;f+s]lts k\|sf/** | **;+s]t** |
| --- | --- | --- | --- |
| F1 | मलाई आफ्नो बच्चालाई स्तनपान गराउन……x'G5 . | **अति कठिन**  **कठिन**  **तटस्थ**  **सजिलो**  **अति सजिलो** | !  @  #  $  % |
| F2 | मैले मेरो बच्चालाई स्तनपान u/fpFbf यस्तो cj:yf आइ पर्न सक्छ कि जस्ले स्तनपान गराउनaf6 रोक्न सक्छ . | **धेरै असम्भावना छ**  **असम्भावना छ**  **तटस्थ**  **सम्भावना छ**  **धेरै सम्भावना छ** | !  @  #  $  % |
| F3 | Df ;Fu आफ्नो बच्चालाई आवश्यक परेको बेलामा स्तनपान गराउन सक्छु भन्ने बिश्वास छ . | **धेरै विश्वास छैन**  **विश्वास छैन**  **तटस्थ**  **विश्वास छ**  **धरै विश्वास छ** | !  @  #  $  % |

S1. Table 11. Breast feeding descriptive norm score (English)

| SN | Statements | Options | Score |
| --- | --- | --- | --- |
| G1 | How did your siblings grown up, were they breastfed or formula fed? | Always bottle fed  Mostly bottle fed  Both breastfed and formula feed  Mostly breastfed  Always breastfed | 1  2  3  4  5 |
| G2 | When I was a baby, my mother …. me. | Bottle fed  Both breastfed and formula fed  Breastfed | 1  2  3 |
| G3 | Have you seen other mothers breastfeeding? | No, never  Sometimes  Always  Daily | 0  1  2  3 |

S1. Table 12. Breast feeding descriptive norm score (Nepali)

| **qm= ;=** | **k\|Zg** | **;f+s]lts k\|sf/** | **cFs** |
| --- | --- | --- | --- |
| G1 | तपाइँका भाइबहिनीहरूn] खाना tyf :tgkfg s] u/]/ x'ls{P, lgDg विकल्पहरू dWo] s'g rfxL ldN5 < | सबै बोतल फेड / थाहा छैन  लगभग बोतल फेड  सबै मध्ये प्रत्येक  लगभग स्तनपान  सबै स्तनपान | !  @  #  $  % |
| G2 | तपाईं बच्चा हुँदा तपाईंको आमाले तपाईंलाई कसरी खुवाउनु भयो< | बोतल फेड  मिस्रीत  स्तनपान | !  @  # |
| G3 | तपाईंले अरु आमाले स्तनपान गरेको देVgभएको छ < | छैन  कहिलेकाँही  दिनदिनै  बारम्बार | )  !  @  # |

S1. Table 13. Breast feeding moral norm score (English)

| **SN** | **Statements** | **SD** | **D** | **N** | **A** | **SA** |
| --- | --- | --- | --- | --- | --- | --- |
| H1 | I will find myself correct when I breastfeed my baby. | 1 | 2 | 3 | 4 | 5 |
| H2 | It is against my principle to breastfeed my baby. | 1 | 2 | 3 | 4 | 5 |
| H3 | Have you seen other mothers breastfeeding? | 1 | 2 | 3 | 4 | 5 |
| H4* | I will find myself correct when I bottle feed my baby. | 1 | 2 | 3 | 4 | 5 |

S1. Table 14. Breast feeding moral norm score (Nepali)

| **qm= ;=** | **k\|Zg tyf c+s** | **अति असहमत** | **असहमत** | **तटस्थ** | **सहमत** | **अति सहमत** |
| --- | --- | --- | --- | --- | --- | --- |
| H1 | म मेरो बच्चालाई स्तनपान गर्न सही महसुस गर्नेछु'. | ! | @ | # | $ | % |
| H2 | मलाई मेरो बच्चालाई बोटल दुध खुवाउदा म आफुँलाई दोषी महसुस गर्नेछु . | ! | @ | # | $ | % |
| H3 | मेरो बच्चालाई बोटल दुध खुवाउनु भनेको मेरो सिद्धान्तहरू विरुद्ध हो . | ! | @ | # | $ | % |
| H4* | मेरो बच्चालाई बोटल दुध खुवाउदा मैले सही महसुस गर्नेछु . | ! | @ | # | $ | % |

S1. Table 15. Breast feeding self-identity score (English)

| **SN** | **Statements** | **SD** | **D** | **N** | **A** | **SA** |
| --- | --- | --- | --- | --- | --- | --- |
| I1 | Breasfeeding is an important part of who I am. | 1 | 2 | 3 | 4 | 5 |
| I2 | I find myself poor when I can’t breastfeed. | 1 | 2 | 3 | 4 | 5 |
| I3* | I don’t like others identify me as a breastfeeding mother. | 1 | 2 | 3 | 4 | 5 |
| I4 | I don’t like others identify me as a bottle-feeding mother. | 1 | 2 | 3 | 4 | 5 |

S1. Table 16. Breast feeding self-identity score (Nepali)

| **qm= ;=** | **k\|Zg** | **अति असहमत** | **असहमत** | **तटस्थ** | **सहमत** | **अति सहमत** |
| --- | --- | --- | --- | --- | --- | --- |
| I1 | बच्चालाई स्तनपान गराउनु d sf] x'F eGg] af/]sf] Ps महत्तोपूर्ण भाग हो . | ! | @ | # | $ | % |
| I2 | बच्चालाई स्तनपान गराउन नपाउँदा d दु:खL x'G5' . | ! | @ | # | $ | % |
| I3* | मलाई मन पर्दैन कि अरु मान्छेले मलाई स्तनपान गर्ने महिलाको रुपम देखोस् . | ! | @ | # | $ | % |
| I4 | मलाई मन पर्दैन कि अरु मान्छेले मलाई बोटल दुध खुवाउने महिलाको रुपम देखोस् . | ! | @ | # | $ | % |

*Scores needs to be reversed before summation.
