# Supplementary material for: Self-identity explains better breastfeeding intention of ethnic pregnant mothers of Western Nepal: Extending the theory of planned behavior
Source: PLoS One. 2024 Oct 11;19(10):e0312010. doi: 10.1371/journal.pone.0312010 (PMC11469527; doi:10.1371/journal.pone.0312010)
Supplement: Attachment — Submitted filename: Response to reviewers_old.docx [file pone.0312010.s003.docx]

To,

The Editor/Handling Editor/Reviewers,

PLOS ONE

**Subject: Rebuttal Letter**

Dear Sir,

Greetings!

Thank you so much for your time to rigorous review and providing the important comments and suggestions! **We have addressed all the comments provided as illustrated in the table (below)**

We are submitting the revised manuscript entitled, “Self-identity explains better breastfeeding intention of ethnic pregnant mothers: Extending the theory of planned behavior”. Along with this, we have also attached the “Track-changed file” and the “Clean manuscript”.

**Table. Comments and**

| **SN** | **Heading/Sub-heading** | **Editor’s Comments** | **Revision/Rebuttals/Reason** |
| --- | --- | --- | --- |
| 1 | Format requirement | Please ensure that your manuscript meets PLOS ONE's style requirements, including those for file naming. | Thank you! We reviewed main-text, funding statement, CoI, Acknowledgement and revised as per ‘Title’ and ‘Body formatting guidelines’ of the PLOS ONE. |
| 2 | Funding information | Please note that funding information should not appear in any section or other areas of your manuscript. We will only publish funding information present in the Funding Statement section of the online submission form. Please remove any funding-related text from the manuscript. | Funding information has been removed from the manuscript part. |
| 3 | Funding statement | Thank you for providing the following Funding Statement:  "This research was conducted with partial financial support from School of Health and Allied Sciences (SHAS) of Pokhara University, Nepal, however, the authorities had no role in design, data collection, analysis and manuscript preparation. CA and RD are permanent faculty members of SHAS, and were benefitted for a week-long leave for data collection and study related management. KG, BB and RB were undergraduate research students of study, without being affected in their own research projects. The authors declare that there are not any other commercial or financial relationships that could be construed as a potential conflict of interest."  We note that one or more of the authors is affiliated with the funding organization, indicating the funder may have had some role in the design, data collection, analysis or preparation of your manuscript for publication; in other words, the funder played an **indirect role** through the participation of the co-authors.  If the funding organization did not play a role in the study design, data collection and analysis, decision to publish, or preparation of the manuscript and only provided financial support in the form of authors' salaries and/or research materials, please **review your statements relating to the author contributions**, and **ensure you have specifically and accurately indicated the role**(s) **that these authors had in your study in the Author Contributions section** of the online submission form. Please make any necessary amendments directly within this section of the online submission form.  Please also update your Funding Statement to include the following statement: “The funder provided support in the form of salaries for authors [insert relevant initials], but did not have any additional role in the study design, data collection and analysis, decision to publish, or preparation of the manuscript. The specific roles of these authors are articulated in the ‘author contributions’ section.”  If the funding organization did have an additional role, please state and explain that role within your Funding Statement.  Please also provide an updated Competing Interests Statement declaring this commercial affiliation along with any other relevant declarations relating to employment, consultancy, patents, products in development, or marketed products, etc.  Within your Competing Interests Statement, please confirm that this commercial affiliation does not alter your adherence to all PLOS ONE policies on sharing data and materials by including the following statement: "This does not alter our adherence to PLOS ONE policies on sharing data and materials.” (as detailed online in our guide for authors  http://journals.plos.org/plosone/s/competing-interests).  If this adherence statement is not accurate and there are  restrictions on sharing of data and/or materials, please state these. Please note that we cannot proceed with consideration of your article until this information has been declared. | **▪Funding statement is revised as:**  “This research was funded from School of Health and Allied Sciences (SHAS) of Pokhara University, Nepal (Faculty Research Grant no. 650-2074/2075), The funder provided a support in the form of paid leaves of two weeks for data collection [CA], but did not have any additional role in the study design, data analysis, decision to publish, or preparation of the manuscript. The specific roles of these authors are articulated in the ‘author contributions’ section.”  **▪Author contribution revised as**  Conceptualization (CA, YRU, RD, KG); Data Curation (CA, KG, BB, RB); Formal Analysis (CA, KG, CLR, LBK, PT, YRU);Fund Acquisition (CA); Investigation (CA, KG, BB, RB); Methodology (CA, RD, PT, CLR), Project Administration (CA, KG, RB); Resources (CA, KG, RB); Software (CA, KG, LBK); Supervision (RD, CA); Validation (CLR); Visualization (CA); Writing- Original Draft Preparation (All Authors); Writing- Review & Editing (CA, RD, CLR, PT)  **▪Funding organization’s role is included in the funding statement**  **▪Competing interest is updated as:**  CA and RD are faculty members under SHAS. KG, BB and RB were undergraduate final semester research students under CA. This does not alter our adherence to PLOS ONE policies on sharing data and materials. |
| 4 | Data Availability Statement | Please provide a complete Data Availability Statement in the submission form, ensuring you include all necessary access information or a reason for why you are unable to make your data freely accessible. If your research concerns only data provided within your submission, please write "All data are in the manuscript and/or supporting information files" as your  Data Availability Statement. | **▪**Revised as “All data are in the manuscript and supporting information files.” |
| Reviewer’s comments (Reviewer #1) | | | |
| 1 | Title | Title- this study was conducted in Nepal, reader should know from the title, and they start to assume the context. Currently, I knew it was about Nepal when I reached to mid of introduction section. | We are thankful to the reviewer. The title is revised as “Self-identity explains better breastfeeding intention of ethnic pregnant mothers of Western Nepal: Extending the theory of planned behavior” |
| 2 | Abstract | Brief context (one sentence) is needed about the problem in the abstract section. | Sorrily I would like to reiterate that it has been mentioned as “…Although effects of additional theoretical constructs like self-identity, moral norm, and descriptive norm, and socio-demographic variables have been explored in the literature, their roles are not clear. Similarly, such exploration among the ethnic and low-income mothers are even scarce…” Kindly suggest further if not sufficient. |
| 3 | Introduction | Readers are left to assume many things in the introduction section- please elaborate it contextualizing breast feeding, and available literature in the topic in Nepal, culture, practices, and trends on it. Secondly, it is necessary to detailed in PTB, thirdly, research gaps, significance and objective. Elaborate it to at least two pages | ▪Thank you for this comment, especially, the need of theoretical explanation of TPB, it is found elsewhere (Ajzen I. The theory of planned behavior. Organizational behavior and human decision processes. 1991 Dec 1;50(2):179-211.), including its evidence synthesis in breast feeding (<https://link.springer.com/article/10.1007/s10995-018-2453-x>), and BF testing in TPB rather than theoretical description, is our study aim.  ▪We agree that culture is obviously related to the constructs and practices of BF intention and behavior, however, this will be out of the scope of our study. Moreover, we are concerned only with cross-sectional empirical evidence, the trend, we assume, would also be out of the scope of this study. However, agreed **these are important, we included in limitations: The trend and cultural influences …**  ▪PLOS guideline does not intricate the limitations regarding number of words or pages for chapters/headings provided that the research gap, objectives and significance are clearly mentioned (https://journals.plos.org/plosone/s/file?id=ba62/PLOSOne_formatting_sample_title_authors_affiliations.pdf).  ▪Sorrily, we have clearly mentioned the BF and BFI, their theoretical and empirical gaps, including in Nepalese contextual practices as…  “Breastfeeding (BF) promotion is the most cost-effective intervention to advance mother-child health(1). BF, including early initiation of it (EIBF), not only reduces infant and under-five mortalities but also plays key roles in maternal health promotion by protecting mothers against postpartum bleeding, breast and ovarian cancer, and osteoporosis (2,3). BF should be initiated as early as and beneficial when continued as long as possible, for which a mother should be prepared before a child is delivered, when only a breastfeeding intent (BFI), not the actual behavior, can be assessed. Even more importantly, mothers, mainly nulliparous, do not adhere to BF, mainly EIBF(4). In the same predicament, only 80% of newborn babies were found to be breastfed within one hour of delivery in the Nepalese context (5).”….  ▪Kindly, we wanted to clarify in the study that we were more concerned towards the cognitive and social process (social psychological) variables of BFI and more delineated towards this. |
| 4 | Methods | Methods section also needs more detail explanation- research context, selection of participants, variables, data analysis model, results section reported beta coefficient but that was not described in the methods section. Very importantly, there is no data how four constructs- BFA, PBFC, BFSI, and BFSN were created (as these were presented quantitatively) | ▪We are thankful for this comment. We added the context, selection of pregnant mothers who completed their first trimesters.  ▪ Thnak you for the comment. For this, we added a Supplemental file (S1. Table 1-16_Constructs and measurement). We also added in the manuscript, with a separate sub-heading, Scoring of constructs, and added text “We assessed BFA with 12 statements, BFSN with two, PBFC with three, BFI with five, BFSI with four, BFK with seven, and BFDN and BFMN each with three statements. All the statements were assessed in 5-point Likert scales, except for BFK and BFDN, with possible value ranges of 12-60, 2-10, 3-15, 5-25, 4-20, and 3-15 for BFA, BFSN, PBFC, BFI, BFSI, and BFMN, respectively. BFK had seven statements with value ranges of 0-1 and 1-3 (possible range; 2-11), whereas BFDN had three statements with value ranges of 0-3, 1-3 and 1-5. Possible values of both BFK and BFDN were 2-11. We dummied the variables with median values for modelling (S1.Table 1-16).”  ▪ Kindly would like to reiterate that we have mentioned the stepwise logistic regression model and various related parameters. |
| 5 | Results | Table can be included in the findings section- that reads easy with the text | Thank you for this comment, will be managed further. |
| 6 | Discussion | Discussion section lacks contextualities of findings relating with policy and programs of Nepal. Interpretation should be  focused comparing with other studies in Nepal. | Dear Reviewer, thank you for the comment. We added five more recent refs (including in limitations), after which there are 23 refs in discussion, which clearly shows the direct and mediating effects of theoretical constructs including the additional-BFSI and income, we have enriched the discussion. Since this is more a theory testing the mediating effects of BFSI and income, in all the theoretical constructs of TPB in breastfeeding (as we have already mentioned also) rather than directly related with a policy or program, and contextualizing. The study findings would possibly guide the policy or program. If you want to make it differently with policy or program to be related, we request to further clearance in this regard. Moreover, since this is a theory-driven research, and local and contextualized researches are very scarce in Nepalese context. |
| **Reviewer # 2** | | | |
| 1 | Format | It has an ethical statement clearly stated | Thank you for your complement. |
| 2 | Methods/Discussion | It is better if the document adds operational definitions and discusses all possible reasons for an association. | Thanks for this comment. We have added with a separate sub-heading under “Scoring of constructs” and added the text, as mentioned above (Reviewer #1, Comment 4) |

Hoping for a positive response from your side,

**Chiranjivi Adhikari, MPH, PhD candidate**

Emails: chiranadhikari@gmail.com/cadhikari@iiphg.org

**Title, authors, word counts, supports and acknowledgements**

**Manuscript type**	: Original

**Title**	: Self-identity explains better breastfeeding intention of ethnic pregnant mothers in western Nepal: Extending the theory of planned behavior

**Short title	:** Self-identity, theory of planned behavior and breastfeeding intention

**Name(s) of the author(s) and affiliations:**

Chiranjivi Adhikari ^1,2*^; Rojana Dhakal^1^; Kapil Giri ^1^; Bidhya Bhandari^1^; Rameshor Baral^1^; Krishna Prasad Pathak^3^; Lal Bahadur Kunwar^4^; Poshan Thapa^5^; Yadu Ram Upreti^6^ ;Khem Narayan Pokharel^7^; and Chhabi Lal Ranabhat^8^

^1^Pokhara University, School of Health and Allied Sciences, Pokhara, Kaski, Nepal

^2^Indian Institute of Public Health Gandhinagar (IIPHG), Gandhinagar, India

^3^Department of Preventive Medicine, Federal University of São Paulo (UNIFESP), Sao Paulo, Brazil

^4^Medic Mobile, Kathmandu, Nepal

^5^School of Population Health, University of New South Wales, Sydney, Australia

^6^Central Dept. of Health and Physical Education, Tribhuvan University, Kathmandu, Nepal

^7^Development and Research Service International, Khumaltar, Lalitpur, Nepal

^8^Department of Health Promotion and Administration, Eastern Kentucky University, USA

***Name and postal address of corresponding author:**

Mr. Chiranjivi Adhikari

Pokhara University, School of Health and Allied Sciences, Pokhara-30, 33700, Nepal

Email: [chiranadhikari@gmail.com](mailto:chiranadhikari@gmail.com)

**FUNDING**

CA received a research grant from School of Health and Allied Sciences (SHAS) of Pokhara University under the faculty grant no. 650-2074/2075.

**Email address(es) of all authors**

Mr. Chiranjivi Adhikari, Lecturer, MPH, PhD candidate, SHAS, Pokhara University (PU), Pokhara, Nepal (Email: chiranadhikari@gmail.com)

Ms. Rojana Dhakal, Lecturer, MSc, PhD candidate, SHAS, PU ([rojanabuddhi2@gmail.com](mailto:rojanabuddhi2@gmail.com))

Mr. Kapil Giri, SHAS, PU ([girikapil752@gmail.com](mailto:girikapil752@gmail.com))

Ms. Bidhya Bhandari, SHAS, PU ([biddhyabhandari1625@gmail.com](mailto:biddhyabhandari1625@gmail.com))

Mr. Rameshor Baral, SHAS, PU ([ramubaral57@gmail.com](mailto:ramubaral57@gmail.com))

Dr. Krishna Prasad Pathak, Lalitpur, Nepal ([pathakrishna37@gmail.com](mailto:pathakrishna37@gmail.com))

Mr. Lal Bahadur Kunwar, Medic Kathmandu, Nepal ([edge.kunwar@gmail.com](mailto:edge.kunwar@gmail.com))

Dr. Poshan Thapa, UNSW, Sydney, Australia ([thapaposhan2009@gmail.com](mailto:thapaposhan2009@gmail.com))

Dr. Yadu Ram Upreti, Tribhuvan University, Kathmandu, Nepal (yaduram.upreti@tucded.edu.np)

Dr. Khem Narayan Pokhrel, Lalitpur, Nepal (Email: pokhrelkhem.irh@gmail.com)

Dr. Chhabi Lal Ranabhat (Email: [chhabir@gmail.com](mailto:chhabir@gmail.com))

**No. of pages and word count details**

Total number of pages (abstract only): 1

Total number of words (abstract only, including keywords): 322

Total number of pages (text excluding the references, tables and abstract): 7

Total number of words (text excluding the references, tables and abstract): 2308

Total number tables: 5

Total number of figures: 2

Total number of references: 35

Total number of supplemental files: 2 (1 data set; 1 table of constructs)
