# Supplementary material for: Self-identity explains better breastfeeding intention of ethnic pregnant mothers of Western Nepal: Extending the theory of planned behavior
Source: PLoS One. 2024 Oct 11;19(10):e0312010. doi: 10.1371/journal.pone.0312010 (PMC11469527; doi:10.1371/journal.pone.0312010)
Supplement: Attachment — Submitted filename: Response to editor.CA_etal_SI.TPBinBFI.docx [file pone.0312010.s004.docx]

Date: 25^th^ Sep, 2024

To,

Prof. Jennifer Yourkavitch

The Academic Editor

& Journal Office

PLOS ONE

**Subject: Re-Response to the comments for the revised manuscript entitled, “Self-identity explains better breastfeeding intention of ethnic pregnant mothers of Western Nepal: Extending the theory of planned behavior”.**

Dear Jennifer,

And Journal Office, PLOS

Greetings!

Thank you so much, again for your time that you provided for reviewing and also the important comments and suggestions! **We have addressed all the comments in the table (below)**

We are submitting a further revised manuscript, with figures revised as suggested from the PACE. We also checked all the references in the below (given) website for ‘retraction check’ and again, this time, in another online platform—the grammar check, and paraphrasing (in some complex paragraphs and sentences). We also checked thoroughly again, as you suggested for the language, coherence, meaning and syntax. As instructed, along with this, we have also attached the “Track-changed file” and the “Clean manuscript”.

**Table. Comments and Revision**

| **Comment No** | **Heading/Sub-heading** | **Editor’s Comments** | **Revision/Rebuttals/Reason** |
| --- | --- | --- | --- |
| **Comments by Academic Editor on Sep 05, 2024** | | | |
| 1 | Journal requirement | Please review your reference list to ensure that it is complete and correct. If you have cited papers that have been retracted, please include the rationale for doing so in the manuscript text, or remove these references and replace them with relevant current references. Any changes to the reference list should be mentioned in the rebuttal letter that accompanies your revised manuscript. If you need to cite a retracted article, indicate the article’s retracted status in the References list and also include a citation and full reference for the retraction notice. | Thank you, for making aware about the journal requirement. We checked all the references, 1-52, for completeness. For retraction check, we visited **The Retraction Watch Database** (<http://retractiondatabase.org/RetractionSearch.aspx?AspxAutoDetectCookieSupport=1#?AspxAutoDetectCookieSupport%3d1%26ttl%3dInfant%2bfeeding%2binformation%252c%2battitudes%2band%2bpractices%253a%2ba%2blongitudinal%2bsurvey%2bin%2bcentral%2bNepal>) mainly, and also in the “PUBPEER” in some selected cases, and in some, when the ‘Watch database’ was not giving clear retraction records.  We did not find any of our references ‘retracted’. |
| 2 | Whole manuscript | Please check again for correct English grammar throughout the manuscript. For example, in the Methods section of the Abstract, you want to say "six purposively selected..." rather than the current phrasing. | Thank you for this point. We corrected this one, and again, for the whole document, we checked in online platform, for grammar check (*https://quillbot.com/grammar-check*), by each sentence. We also checked manually, line-by-line for the errors. |
| 3 | Methods | Spell out all of the acronyms at the first usage (under "Scoring of Constructs"). Please also put the footnote about family type in Table 1, where the distinction first appears. | Thank you for the comment!  We have done with these all.  women (85.8%) receive the first ANC services within four months of pregnancy. As mothers come generally to the HCFs several weeks after their amenorrhea, it is difficult to get enrolled before their first trimester.  we enrolled conveniently among from those who completed their first trimester. This has been described briefly in methods section. |
| 4 | Methods | Also put the footnote about family type in Table 1, where the distinction first appears. | Sorry for this typo error, as earlier it was supposed to be done. Now addressed, and checked once again. |
| 5 | Figures | Checking figures in the “PACE” platform for journal compatibilities | Thank you! We checked without any difficulty, and as suggested, we are now uploading the ‘produced’ ones. |
| **Comments by Journal office on Sep 24, 2024** | | | |
| 1 | Files | We note that several of your files are duplicated on your submission. Please remove any unnecessary or old files from your revision, and make sure that only those relevant to the current version of the manuscript are included. | Thank you. I have removed the older correction/trackchanged and other files, and tetained the recent files. |
| 2 | Title | Please amend the title either on the online submission form or in your so that they are identical. | We are extremely sorry, to update in cover letter, now checked and corrected in online, manuscript and other submitted documents. Kindly let us remind if still there is discrepancy. |
| 3 | Figure | Please remove your figures/ from within your manuscript file, leaving only the individual TIFF/EPS image files.  These will be automatically included in the reviewer’s PDF | Thank you. We have corrected accordingly. |
| 4 | Figure | Please include a separate legend for each figure in your manuscript. | The figure legend has been been separated from the main figure and kept where they occur in the text (manuscript). |
| **Comments by Journal office on Sep 25, 2024** | | | |
| 1 | Title | Please amend the title either on the online submission form or in your so that they are identical. | Now, checked for each document, and corrected as “Self-identity explains better breastfeeding intention of ethnic pregnant mothers of Western Nepal: Extending the theory of planned behavior” |

Hoping for a positive response from your side,

Thank you once again for your important contribution!

**Chiranjivi Adhikari, MPH, MED, PhD candidate**

Emails: chiranadhikari@gmail.com/cadhikari@iiphg.org
